# Chloroquine efficacy for *Plasmodium vivax* malaria treatment in southern Ethiopia

**DOI:** 10.1186/s12936-015-1041-4

**Published:** 2015-12-24

**Authors:** Sisay Getachew, Kamala Thriemer, Sarah Auburn, Adugna Abera, Endalamaw Gadisa, Abraham Aseffa, Ric N. Price, Beyene Petros

**Affiliations:** Addis Ababa University, Addis Ababa, Ethiopia; Armauer Hansen Research Institute, Addis Ababa, Ethiopia; Global and Tropical Health Division, Menzies School of Health Research, Charles Darwin University, PO Box 41096, Casuarina, Darwin, NT 0811 Australia; Centre for Tropical Medicine and Global Health, Nuffield Department of Medicine, University of Oxford, Oxford, UK

**Keywords:** Malaria, Vivax, Chloroquine, Ethiopia, Resistance

## Abstract

**Background:**

Chloroquine (CQ) is the first-line treatment for vivax malaria in Ethiopia, but there is evidence for its declining efficacy. Defining the extent and regional distribution of CQ resistance is critical to ensure optimal treatment guidelines. This study aimed to provide data on the therapeutic efficacy of CQ against *Plasmodium vivax* malaria in southern Ethiopia.

**Methods:**

Patients with *P. vivax* mono-infection aged between 8 months and 65 years were enrolled in a clinical efficacy trial. The study was conducted at four sites in southern Ethiopia. Study participants were treated with a supervised course of CQ (25 mg/kg over three consecutive days), followed by weekly blood film examination and clinical assessment for 28 days. CQ blood concentrations were not assessed. The primary endpoint was the risk of failure at 28 days by survival analysis.

**Results:**

Between May 2010 and December 2013, 288 patients were enrolled in the study (n = 89 in Shele, n = 52 in Guba, n = 57 in Batu and n = 90 in Shone). Baseline characteristics varied significantly between sites. In total 34 (11.8 %) patients were censored during follow up (five with *Plasmodiumfalciparum* parasitaemia and 29 lost to follow up). Two (0.7 %) patients experienced early treatment failure and 23 (8 %) late treatment failure. The overall risk of recurrence by day 28 was 9.4 % (95 % CI 6.4–13.6 %) with site-specific estimates of 3.8 % (95 % CI 1.2–11.3) for Shele, 21.9 % (95 % CI 12.2–36.1) for Guba, 5.9 % (95 % CI 1.9–17.3) for Batu and 9.2 % (95 % CI 4.5–17.6) for Shone.

**Conclusion:**

There is evidence of reduced CQ efficacy across three of the four study sites, with the degree of resistance severe enough in Guba to suggest that review of treatment policy may be warranted.

## Background

An estimated 2.85 billion people live at risk of *Plasmodium vivax* infection worldwide [1, 2] with the number of annual infections ranging from 19 to 240 million cases [3]. The greatest burden of disease is found in South and Southeast Asia, although 10–20 % of all *P. vivax* malaria cases occur in Eastern and Southern Africa [1]. Over the last decade *Plasmodium falciparum* transmission has declined more rapidly than *P. vivax* in endemic regions and *P. vivax* is now emerging as the dominant species in many co-endemic areas [3, 4].

In Ethiopia, *P. vivax* accounts for approximately 40 % of all malaria infections [5]. Historically, there have been an estimated 10 million clinical malaria cases annually, although numbers have reduced substantially in the past decade. Since 2005, Ethiopia has scaled-up one of the largest and most ambitious malaria control programmes in Africa. The programme is designed to support the country‘s Health Sector Development Plan (HSDP), and the national child survival strategy, in order to reduce under-five mortality rates by two thirds by 2015 [6].

In most parts of the world chloroquine (CQ) remains the first-line treatment for *P. vivax* malaria. However, the emergence of drug resistance has compromised its use [7, 8] in several regions. Since 2004, artemether–lumefantrine (AL) has been used for uncomplicated falciparum malaria in Ethiopia, but CQ is still used for vivax malaria [5]. Several studies have documented reduced CQ sensitivity against *P. vivax* in different regions of Ethiopia [9–11], although this appears sporadic, since other studies show sustained efficacy [12]. The evidence suggests an emerging pattern of declining CQ susceptibility in this region that will undermine control efforts for both falciparum and vivax malaria and emphasizes the need for ongoing surveillance of CQ efficacy.

The objective of the current clinical study was to provide an update on the therapeutic efficacy of CQ against *P. vivax* malaria in four locations in southern Ethiopia.

## Methods

### Study sites and participants

**Fig. 1:**
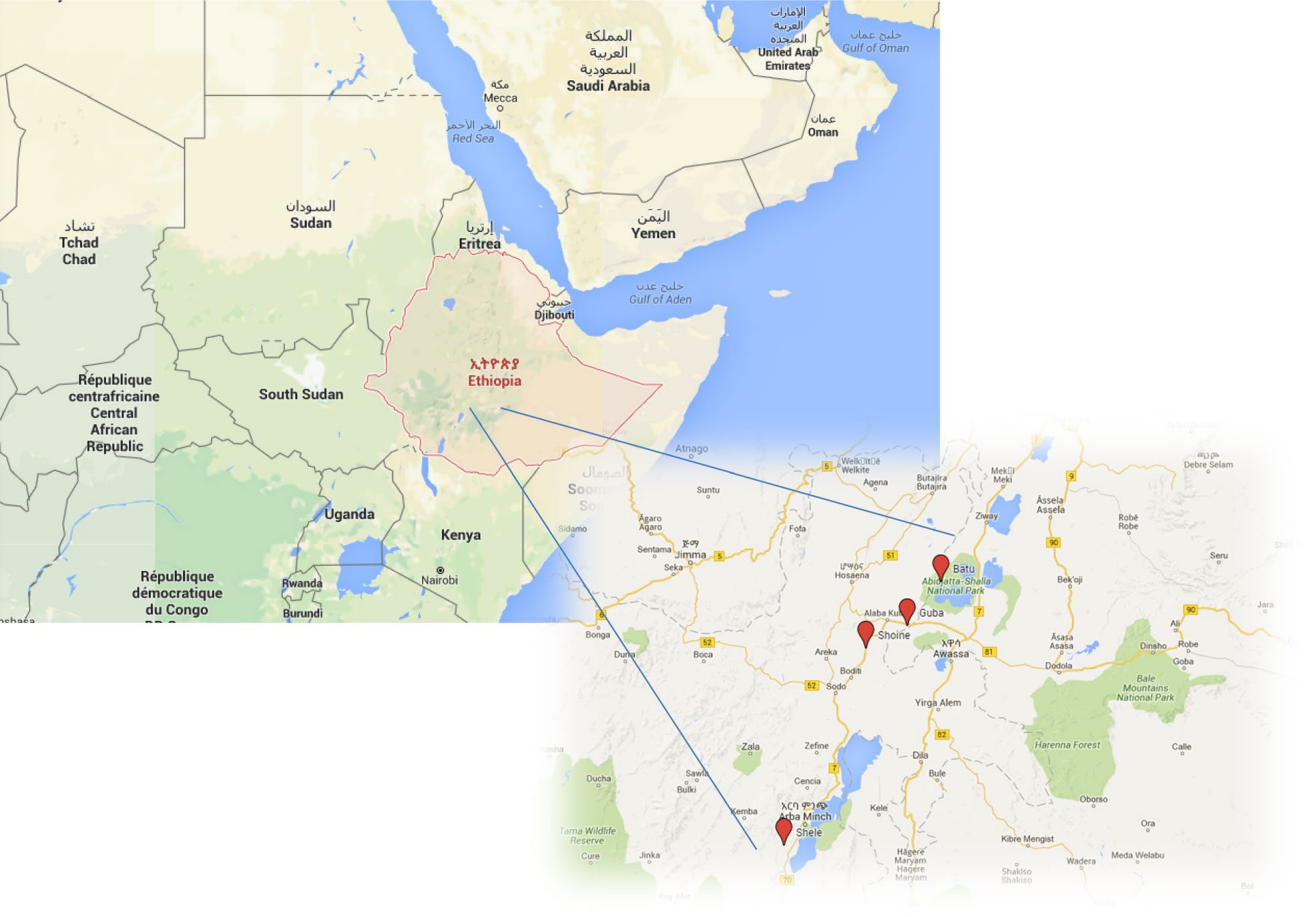
Map of study sites

The study was conducted in Southern Ethiopia, at health centres located in Shele in Arba Minch Zuria district, Guba in Halaba district, Batu in Adami Tulu Jido Kombolcha district and Shone in Eastern Badawacho district (Fig. 1) between May 2010 and December 2013. Patients attending outpatient clinics with signs and symptoms consistent with malaria were screened for inclusion into the study. Inclusion criteria were as follows: age >6 months, *P. vivax* mono-infection with parasite densities above 250/µl, an axillary temperature of ≥37.5 °C or history of fever within 48 h of presentation, residence in close proximity to the health centre (i.e. within 10 km radius) and willingness to participate in the study and be followed up for at least 28 days. Written informed consent from the patient or in case of minors from a parent or guardian and assent from children aged 12–17 was obtained. Patients with manifestations of severe malaria as defined by the World Health Organization (WHO) criteria were excluded [13]. Patients were excluded if they had severe malnutrition, were pregnant or had clinically evidence of chronic disease, a history of allergies and/or intolerance to CQ, or taken any anti-malarial or antibiotic treatment within the previous 2 weeks.

### Study design

The study design was based on the standard 28 day follow up survey as defined by the WHO [14]. Patients with uncomplicated *P. vivax* infection were recruited and treated at the respective health centres. Patients were asked to return to the health centre on day 1 and 2 for directly observed treatment. Patients were then assessed at follow up visits on days 3, 7, 14, 21 and 28 or whenever they had signs and symptoms consistent with malaria. Patients failing to come to their scheduled appointments were visited at home.

### Treatment

Quality-assured CQ (KT9363 and BN11747) from the Ethiopian Federal Ministry of Health was used as the study treatment. CQ was administered at 25 mg base/kg body weight divided over 3 days (10 mg base/kg on day 0 and 1 and 5 mg base/kg on day 2). Study participants were observed for 30 min after administration and those vomiting their dose were treated again with the same dose of CQ and observed for an additional 30-min period. Patients failing to respond to CQ therapy were treated with quinine as a second line treatment [5]. Patients with a temperature >38.0 °C were prescribed oral paracetamol. In line with current national treatment guidelines no primaquine was given [5].

### Clinical procedures

A general physical examination was conducted at the enrollment visit. Baseline data on age, sex, weight and height were recorded. Adverse events were recorded at every visit. A finger prick blood sample was taken for microscopy at every follow up visit and any unscheduled visit. Haemoglobin was measured on the day of enrolment and on day 28.

### Laboratory procedures

#### Pregnancy test

All female study participants aged 12 years and older were tested for pregnancy. Urine human chorionic gonadotropin (HCG) dipstick test (ACON laboratories, INC. CA, USA) was used and pregnant women were excluded from the study.

#### Microscopy

Thick and thin blood smears were prepared in duplicate. Blood slides were stained with Giemsa, as described previously [14]. Blood films were read by two independent laboratory technicians from the health centre; in case of discordance a third reading was performed. Parasite densities were recorded for all positive slides. The number of asexual parasites was counted per 200 white blood cells (WBC) and parasitaemia estimated assuming a WBC count of 8000/µl. Gametocytes were counted against 100 fields. Before a slide was considered negative a total of 100 WBCs were counted [14]. A random set of 10 % of the slides were cross checked by another experienced laboratory technician for quality control at the Nazareth Malaria Control Centre.

#### Haemoglobin

One drop of blood was used to measure the haemoglobin concentration on day 0, on the day of recurrent parasitaemia and on day 28 using Hemocue™ (Angelholm Sweden).

### Study endpoints

The primary endpoint of the study was risk of recurrence by day 28 [15]. Treatment failures were categorized as early (ETF), late parasitological failures (LPTF) and late clinical treatment failures (LCTF). ETFs were defined as one of the following: (1) the occurrence of danger signs or severe malaria on day 1, 2 or 3 in the presence of parasitaemia, (2) parasitaemia on day 2 higher than on day 0 irrespective of axillary temperature, (3) parasitaemia on day 3 with axillary temperature ≥37.5 °C or (4) parasitaemia on day 3 ≥25 % of count on day 0. LCTFs were defined as the occurrence of danger signs or severe malaria in the presence of parasitaemia or the presence of parasitaemia on any day between day 4 and day 28 with axillary temperature ≥37.5 °C (or history of fever) in patients who did not previously meet any of the criteria of early treatment failure. LPTFs were defined as the presence of parasitaemia on any day between day 7 and day 28 with axillary temperature <37.5 °C in patients who did not previously meet any of the criteria of early treatment failure or late clinical failure. Patients with no parasitaemia on day 28, irrespective of axillary temperature, and who did not previously meet any of the criteria of early treatment failure, late clinical failure or late parasitological failure were defined as adequate clinical and parasitological response (ACPR) [15]. Secondary endpoints were adjusted efficacy estimates at day 28 and parasite clearance.

### Statistical analyses

Data were double entered into the WHO excel spreadsheet. All statistical analyses were performed using STATA 13 (StatCorp, USA). The Mann–Whitney U test or Kruskal–Wallis method were used for nonparametric comparisons, and Student’s *t* test or one-way analysis of variance for parametric comparisons. For categorical variables percentages and corresponding 95 % confidence intervals (95 % CI) were calculated using Wilson’s method. Proportions were examined using χ^2^ with Yates’ correction or by Fisher’s exact test. The cumulative risk of failure was assessed by survival analysis using the Kaplan–Meier method on a modified intention to treat basis. Anyone lost to follow up or presenting with *P. falciparum* infection was censored on their last day of follow up and regarded as not being treatment failures.

### Ethical consideration

The study protocols were reviewed and approved by the respective Ethical Boards of the Addis Ababa University College of Natural Sciences, Ethiopia (RERC/002/05/2013), the Armauer Hansen Research Institute, Addis Ababa, Ethiopia (AHRI-ALERT P011/10), the National Research Ethics Review Committee of Ethiopia (Ref.no. 3.10/580/06) and the Human Research Ethics Committee of the Northern Territory Department of Health and Menzies School of Health Research, Australia (HREC-13-1942). No participant was enrolled without prior written informed consent.

## Results

### Study profile and baseline characteristics

**Fig. 2:**
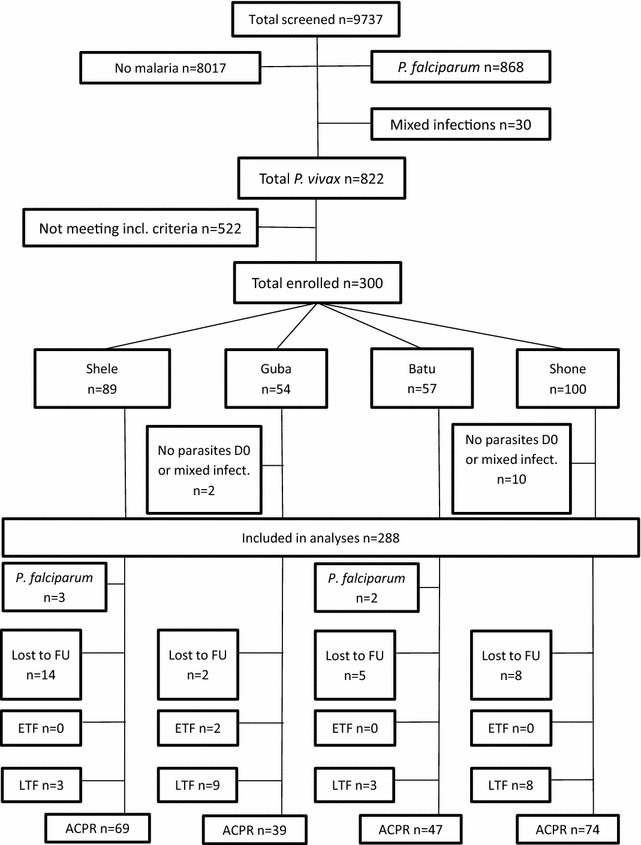
Flow chart of patients in the study

Overall 9737 patients were screened for inclusion into the study, 8017 (82.3 %) had no malaria, 868 (8.9 %) had a falciparum infection and 30 (0.3 %) a mixed infection. Of the 822 (8.4 %) patients with vivax infection 522 (63.5 %) did not meet the inclusion criteria, resulting in 300 (57.5 %) patients enrolled. On cross checking of microscopy 12 (4 %) patients were later confirmed to have either a mixed species infection or no parasites and were, therefore, excluded from the study. A total of 288 patients were included in the analyses (Fig. 2): 89 in Shele, 52 in Guba, 57 in Batu and 90 in Shone.

More than half of the patients were male (56.3 %) and the median age was 8 years (IQR 4–18) with 70.8 % (204/288) patients aged less than 15 years old. There was marked heterogeneity between the study sites in regards to gender, age, body temperature, haemoglobin concentration and parasite density at enrollment (Table 1).

**Table 1:** Baseline data of the study population overall and per site

Variable	Shele	Guba	Batu	Shone	Overall	p value^a^
Total number of patients	89	52	57	90	288	
Gender male (%)	58 (65.2 %)	30 (57.7 %)	23 (40.3 %)	51 (56.7 %)	162 (56.3 %)	0.032
Age						
Median age [IQR] in years	10 [4–18]	12 [4.5–22.5]	12 [6–20]	6 [4–9]	8 [4–18]	0.0022
≤5 year	31 (34.8 %)	16 (30.8 %)	13 (22.8 %)	43 (47.8 %)	103 (35.8 %)	
5 to ≤15 years	26 (29.2 %)	18 (34.6 %)	24 (42.1 %)	33 (36.7 %)	101 (35.1 %)	
>15 years	32 (35.9 %)	18 (34.6 %)	20 (35.1 %)	14 (15.6 %)	84 (29.2 %)	
Body weight						
Median weight [IQR] in kg	22.5 [14–50]	26.5 [15–44]	35 [16.3–50.1]	20 [17–26]	22 [16–45]	0.24
Median mg/kg [IQR] total dose of chloroquine	27.3 [25–30]	27.0 [25–30]	26.7 [23.6–28.8]	31.25 [26.98–37.5]	27.9 [25–30.8]	0.0001
Enrolment clinical parameters						
Mean (SD) temperature °C	37.5 (0.82)	37.6 (1.18)	37.0 (0.74)	37.0 (0.68)	37.3 (0.88)	0.0003
Fever (temp >37.5 °C) N (%)	44 (49.4 %)	20 (38.5 %)	18 (31.6 %)	25 (27.8 %)	107 (37.2 %)	0.019
Geometric mean parasitaemia µl^−1^ [95 % CI]	3112 [1483–3899]	6527 [4709–9046]	1961 [1661–2314]	1751 [1417–2164]	2712 [2387–3082]	0.0001
Gametocyte carriage N (%)	86 (96.7 %)	22 (42.3 %)	5 (8.8 %)	61 (67.8 %)	174 (60.4 %)	0.000
Geometric mean gametocyte density µl^−1^ [95 % CI]	334 [257–435]	99 [66–148.5]	146 [60–355.1]	26 [20.2–34]	114 [90.3–145.8]	0.000
Mean haemoglobin (SD)^b^	10.6 (1.9)	12.1 (1.5)	13.8 (1.7)	14.0 (1.3)	12.5 (2.2)	0.0001
Anaemic (Hb < 10 g/dL) N (%)^b^	44 (49.4 %)	5 (9.8 %)	2 (3.6 %)	0	51 (18.5 %)	0.000

^a^Difference between sites

^b^Hb not recorded in 13 patients

The total median dose CQ administered was 27.9 mg/kg body weight. Two patients received a total dosage below 20 mg/kg: one patient received 18.75 mg from three doses of medication and the other didn’t complete the entire treatment course before leaving the study (total of 10 mg). Overall 5.9 % (17/288) patients vomited their first dose of CQ and 0.3 % (1/288) their second dose.

### Parasite clearance

**Fig. 3:**
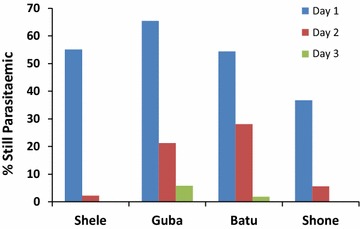
Proportion of patients with parasitaemia

In total, 49 % (141/288) of patients had cleared their peripheral parasitaemia when reviewed on day 1, 88.2 % (254/288) by day 2 and 98.7 % (284/288) by day 3. Whereas patients in Shele and Shone all cleared their parasites by day 3, 5.6 % (3/54) of the patients in Guba and 1.5 % (1/57) in Batu were still parasitaemic at this time (Fig. 3). Patients who were still parasitaemic at day 2 had a significantly higher baseline parasitaemia (geometric mean 5284 µl^−1^ 95 % CI 3525–7918) than those who were aparasitaemic (geometric mean 2446 µl^−1^ 95 % CI 2145–2788; p = 0.0003).

### Efficacy outcomes

**Fig. 4:**
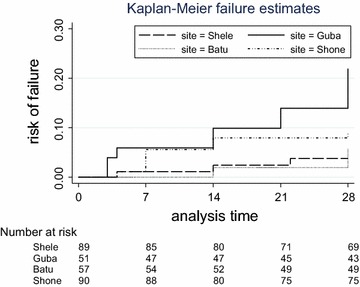
Kaplan–Meier graph with efficacy estimates per site

Of the 288 patients in the evaluable population 29 (10 %) patients were lost to follow up and 5 (1.7 %) had a *P. falciparum* parasitaemia during follow up. Early treatment failures (ETF) occurred in 2 (0.7 %) patients, both due to a slow clearance with patent parasitaemia on day 3 associated with and an axillary temperature >37.5 °C. A late treatment failure (LTF) was observed in 23 (8 %) patients, eight of whom were classified as LCTF and 15 as LPTF. The overall risk of treatment failure at day 28 was 9.4 % (95 % CI 6.4–13.6 %) with site-specific estimates of 3.8 % (95 % CI 1.2–11.3) for Shele, 21.9 % (95 % CI 12.2–36.1) for Guba, 5.9 % (95 % CI 1.9–17.3) for Batu and 9.2 % (95 % CI 4.5–17.6) for Shone (Fig. 4). The median time to recurrence was 14 days (IQR 4–28), the time to recurrence being shorter in adults (median time 5.5 days, IQR 4–7) compared to children (median time 14 days, IQR 7–28).

Although the time to recurrence was not correlated significantly with baseline parasitaemia, patients failing treatment had a significantly higher baseline parasitaemia (geometric mean 5434 µl^−1^; 95 % CI 3238–9118) compared to those successfully treated (2539 µl^−1^; 95 % CI 2230–2890; p = 0.002). Patents with a baseline parasitaemia greater or equal than to 6234 µl^−1^ (the 75th percentile), had an odds ratio (OR) for treatment failure of 3.75 (95 % CI 1.62–8.65; p = 0.02). Patient failing the treatment were also significantly younger than those successfully treated [mean age of 6.1 (SD 6.4) years compared to 12.8 (SD 11.0) years; p = 0.003], and this remained significant after controlling for baseline parasitaemia. There was no difference in the total dose per kilogram administered in patients who failed treatment and those who were treated successfully. There were a total of 18 (6.3 %) patients who vomited any dose of CQ. From those, only one (5.5 %) had a late treatment failure compared to 8.1 % (22/270) in those who did not vomit (p = 0.69).

### Haemoglobin

The mean haemoglobin concentration at baseline was 12.5 g/dL (95 % CI 12.3–12.78) and 12.5 g/dL (95 % CI 12.3–12.8) at day 28, the risk of anaemia (Hb < 10 g/dL) being 18.5 % (51/175) and 16.4 % (35/214) at baseline and at day 28 respectively. Haemoglobin was significantly correlated with baseline parasite density (rho = −0.19, p = 0.0017), and also with age (rho = 0.11, p = 0.058). The risk of anaemia varied significantly between sites being greatest in Shele (49.4 %) (Table 1).

## Discussion

The clinical efficacy study in four locations in southern Ethiopia show evidence of emerging CQ resistance in the region, although there was marked heterogeneity in the risk of treatment failure between the study sites. The national treatment guidelines in Ethiopia recommend CQ for treatment of vivax malaria, although evidence of declining efficacy has raised significant concerns amongst local policy makers [9–11, 16–19]. In a recent study in Halaba, southern Ethiopia, 13.8 % (11/80) of patients had a recurrent *P. vivax* infection by day 28 [18], similar to the results in Shone which is approximately 30 km away.

Parasite recurrence can arise from either parasite resistance to what should be a curative treatment regimen or inadequate treatment such as that from malabsorption, vomiting or poor drug quality, resulting in subcurative drug concentrations in the peripheral blood. Blood concentrations of CQ were not measured in this study, so it is not possible to rule out whether sub-therapeutic drug levels contributed to poor treatment outcome, which is a limitation of this study. However it is reassuring that all patients received a total dose of CQ greater than 27 mg/kg, suggesting that suboptimal dosing was unlikely to have been a contributing factor. Whilst poor absorption, particularly for the initial doses in febrile patients, may contribute to some treatment failure it is unlikely to have caused more than 5 % recurrences [7].

Recurrent *P. vivax* parasitaemia may be due to recrudescence of the same parasite, relapse from the reactivation of a dormant liver stage or a new infection. Relapses can occur as early as 21 days following initiation of treatment. Recrudescent infections can occur late (up to 63 days); although the greater the degree of resistance the earlier the recurrence is likely to occur. Whereas genotyping of polymorphic loci is undertaken routinely in *P. falciparum* drug efficacy trials to distinguish new infections from true parasite recrudescence [20], in *P. vivax* the interpretation of molecular typing is less informative. Heterologous paired isolates from pre- and post-treatment help to rule out true recrudescence, however homologous infection can be due to either recrudescence or relapse [21–23]. Importantly since CQ has a long terminal elimination, any parasite recurring within 28 days is likely to have done so in the presence of high drug concentrations expected to be above the minimum inhibitory concentration of CQ sensitive isolates (100 nM). As such the day of recurrence is as useful measure of CQ resistance (CQR) in the population.

There was noted heterogeneity in the risk of recurrence between and within sites. Treatment failure was higher in patients who were younger, and had a higher parasitaemia at enrollment. Both of these factors are well recognized as important risk factors for failure in *P. falciparum* clinical trials [24, 25]. High baseline parasitaemia is associated with prolonged parasite clearance, which is a reliable predictor of parasite recrudescence. Previous studies in *P. vivax* have demonstrated a similar relationship [26] suggesting that rapid clearance (95 % by day 2 or 100 % by day 3) may be a useful marker of CQ sensitivity [7]. In the current study, two sites fulfilled these criteria and although CQ efficacy in Shele was adequate (>96.2 %), in Shone there was evidence of emerging CQR (efficacy of 90.8 % 95 % CI 82.4–95.3). The population enrolled in Guba had the lowest efficacy with 21.9 % risk of recurrence, and this site also recorded more than 20 % parasitaemia on day 2 and more than 5 % on day 3 (Fig. 3). This site had also a significantly higher enrollment parasite density compared to the other three sites. The risk of recurrence in Guba suggests significant degree of CQR, however efficacy is likely to have been further compromised by the high levels of parasitaemia at presentation [27].

## Conclusions

Overall this study highlights evidence of clinical treatment failure after CQ most likely due to CQ resistance emerging in three out of four sites in southern Ethiopia. The data supports calls for increased drug resistance monitoring and re-evaluation of treatment guidelines.

## References

[1] Guerra CA, Howes RE, Patil AP, Gething PW, Van Boeckel TP, Temperley WH (2010). The international limits and population at risk of *Plasmodium vivax* transmission in 2009. PLoS Negl Trop Dis.

[2] Price RN, Tjitra E, Guerra CA, Yeung S, White NJ, Anstey NM (2007). Vivax malaria: neglected and not benign. Am J Trop Med Hyg.

[3] WHO (2014). World malaria report 2014.

[4] WHO (2015). Control and elimination of *Plasmodium vivax* malaria: a technical brief.

[5] FMOH (2004). Malaria diagnosis and treatment guidelines for health workers in Ethiopia.

[6] FMOH (2012). National malaria guidelines.

[7] Price RN, von Seidlein L, Valecha N, Nosten F, Baird JK, White NJ (2014). Global extent of chloroquine-resistant *Plasmodium vivax*: a systematic review and meta-analysis. Lancet Infect Dis.

[8] Baird JK (2009). Resistance to therapies for infection by *Plasmodium vivax*. Clin Microbiol Rev.

[9] Tulu AN, Webber RH, Schellenberg JA, Bradley DJ (1996). Failure of chloroquine treatment for malaria in the highlands of Ethiopia. Trans R Soc Trop Med Hyg.

[10] Teka H, Petros B, Yamuah L, Tesfaye G, Elhassan I, Muchohi S (2008). Chloroquine-resistant *Plasmodium vivax* malaria in Debre Zeit, Ethiopia. Malar J.

[11] Ketema T, Bacha K, Birhanu T, Petros B (2009). Chloroquine-resistant *Plasmodium vivax* malaria in Serbo town, Jimma zone, south-west Ethiopia. Malar J.

[12] Hwang J, Alemayehu BH, Reithinger R, Tekleyohannes SG, Takele T, Birhanu SG (2013). In vivo efficacy of artemether–lumefantrine and chloroquine against *Plasmodium vivax*: a randomized open label trial in central Ethiopia. PLoS One.

[13] WHO (2012). Management of severe malaria: a practical handbook.

[14] WHO. Methods for surveillance of antimalarial drug efficacy. Geneva: World Health Organization; 2009. http://whqlibdoc.who.int/publications/2009/9789241597531_eng.pdf. Accessed 5 Mar 2013.

[15] WHO (2003). Assessment and monitoring of antimalarial drug efficacy for the treatment of uncomplicated falciparum malaria.

[16] Schunk M, Kumma WP, Miranda IB, Osman ME, Roewer S, Alano A (2006). High prevalence of drug-resistance mutations in *Plasmodium falciparum* and *Plasmodium vivax* in southern Ethiopia. Malar J.

[17] Yohannes AM, Teklehaimanot A, Bergqvist Y, Ringwald P (2011). Confirmed vivax resistance to chloroquine and effectiveness of artemether–lumefantrine for the treatment of vivax malaria in Ethiopia. Am J Trop Med Hyg.

[18] Ketema T, Getahun K, Bacha K (2011). Therapeutic efficacy of chloroquine for treatment of *Plasmodium vivax* malaria cases in Halaba district. South Ethiopia. Parasit Vectors.

[19] Mekonnen SK, Aseffa A, Berhe N, Teklehaymanot T, Clouse RM, Gebru T (2014). Return of chloroquine-sensitive *Plasmodium falciparum* parasites and emergence of chloroquine-resistant *Plasmodium vivax* in Ethiopia. Malar J.

[20] Snounou G, Beck HP (1998). The use of PCR genotyping in the assessment of recrudescence or reinfection after antimalarial drug treatment. Parasitol Today.

[21] Imwong M, Boel ME, Pagornrat W, Pimanpanarak M, McGready R, Day NP (2012). The first *Plasmodium vivax* relapses of life are usually genetically homologous. J Infect Dis.

[22] Chen N, Auliff A, Rieckmann K, Gatton M, Cheng Q (2007). Relapses of *Plasmodium vivax* infection result from clonal hypnozoites activated at predetermined intervals. J Infect Dis.

[23] Craig AA, Kain KC (1996). Molecular analysis of strains of *Plasmodium vivax* from paired primary and relapse infections. J Infect Dis.

[24] Worldwide Antimalarial Resistance Network ALDISG (2015). The effect of dose on the antimalarial efficacy of artemether–lumefantrine: a systematic review and pooled analysis of individual patient data. Lancet Infect Dis.

[25] WorldWide Antimalarial Resistance Network DPSG (2013). The effect of dosing regimens on the antimalarial efficacy of dihydroartemisinin–piperaquine: a pooled analysis of individual patient data. PLoS Med.

[26] Ratcliff A, Siswantoro H, Kenangalem E, Wuwung M, Brockman A, Edstein MD (2007). Therapeutic response of multidrug-resistant *Plasmodium falciparum* and *P. vivax* to chloroquine and sulfadoxine–pyrimethamine in southern Papua, Indonesia. Trans R Soc Trop Med Hyg.

[27] Dorsey G, Gasasira AF, Machekano R, Kamya MR, Staedke SG, Hubbard A (2004). The impact of age, temperature, and parasite density on treatment outcomes from antimalarial clinical trials in Kampala, Uganda. Am J Trop Med Hyg.

